# Targeting Glial Mitochondrial Function for Protection from Cerebral Ischemia: Relevance, Mechanisms, and the Role of MicroRNAs

**DOI:** 10.1155/2016/6032306

**Published:** 2016-09-29

**Authors:** Le Li, Creed M. Stary

**Affiliations:** ^1^Department of Anesthesiology, Perioperative & Pain Medicine, Stanford University School of Medicine, 300 Pasteur Drive, Stanford, CA 94305-5117, USA; ^2^Department of Anesthesiology, Zhujiang Hospital, Southern Medical University, 253 Industrial Road, Guangzhou, Guangdong Province 510280, China

## Abstract

Astrocytes and microglia play crucial roles in the response to cerebral ischemia and are effective targets for stroke therapy in animal models. MicroRNAs (miRs) are important posttranscriptional regulators of gene expression that function by inhibiting the translation of select target genes. In astrocytes, miR expression patterns regulate mitochondrial function in response to oxidative stress* via* targeting of Bcl2 and heat shock protein 70 family members. Mitochondria play an active role in microglial activation, and miRs regulate the microglial neuroinflammatory response. As endogenous miR expression patterns can be altered with exogenous mimics and inhibitors, miR-targeted therapies represent a viable intervention to optimize glial mitochondrial function and improve clinical outcome following cerebral ischemia. In the present article, we review the role that astrocytes and microglia play in neuronal function and fate following ischemic stress, discuss the relevance of mitochondria in the glial response to injury, and present current evidence implicating miRs as critical regulators in the glial mitochondrial response to cerebral ischemia.

## 1. Introduction

Ischemic stroke remains a leading cause of death and long-term disability worldwide [1]. Despite hundreds of promising preclinical trials demonstrating efficacy of neuron-targeted therapies in animal models of stroke, the only clinical treatment remains early restoration of blood flow with thrombolysis [2]. The failure to translate neuron-targeted approaches to useful clinical therapy suggests that alternative cellular targets in the brain may more effectively coordinate the complex intra- and intercellular signaling cascades that contribute to neuronal injury. Astrocytes comprise the most numerous type of cell in the brain and play a crucial role in neuronal homeostasis both for normal physiologic functioning and in response to cell stress [3]. Microglia coordinate growth and remodeling of the neural network and regulate the neuroinflammatory response to stroke [4, 5]. In both astrocytes and microglia, mitochondria play a central role in determining local neuronal cell fate. Therapeutic strategies aimed at maintaining mitochondrial function in glia following stroke may therefore provide a novel approach to reduce the degree of injury and improve neurobehavioral outcome.

MicroRNAs (miRs) are a class of small noncoding RNAs that regulate gene expression by binding to the 3′ untranslated region (UTR) of target genes and destabilizing or inhibiting their translation [6]. In glia, miRs have been shown to play an important role in the cellular response to ischemic injury (for reviews, see [7–9]). In particular, miRs can alter the expression of proteins that both directly and indirectly modulate glial mitochondrial function. The purpose of this review is to (1) provide an overview of astrocyte and microglia-mediated regulation of neuronal cell function and fate following ischemic injury; (2) discuss the relevance of glial mitochondrial function in response to ischemic injury; (3) review coordination of mitochondrial homeostasis by B-cell lymphoma 2 (Bcl2) and heat shock protein 70 (Hsp70) family members; and (4) present current evidence demonstrating the critical role miRs play in regulating glial mitochondrial function in response to cerebral ischemic injury.

## 2. Glia in Health and in Response to Ischemia

### 2.1. Astrocytes

Neuronal maintenance, neurite outgrowth, and repair of the neuronal network are coordinated by resident astrocytes [10–12]. As an essential component of the neurovascular unit (a dynamic structure also composed of endothelial cells, pericytes, basement membrane, and surrounding neurons), astrocytes control blood circulation, extracellular ion homeostasis, and release of energy substrates and growth factors in the central nervous system. In addition to their role in neuronal housekeeping and protection, astrocytes play a significant role in neurotransmission [11, 13]. Astrocytes are central to synapse formation and stabilization in development and disease [3, 14, 15] and modulate synaptic transmission* via* glutamate uptake [16]. Astrocytes extend many fine branching processes, putting them in direct contact with cell bodies, dendrites, and synaptic terminals, such that an individual astrocyte may contact up to 100,000 neurons [17]. Moreover, astrocytes communicate with adjacent astrocytes* via* intercellular gap junctions to function as a coordinated syncytium [18, 19]. As a consequence, astrocytes actively regulate and organize local and distant synaptic activity, excitability, transmission, and plasticity of the neuronal network [20–23].

**Figure 1:**
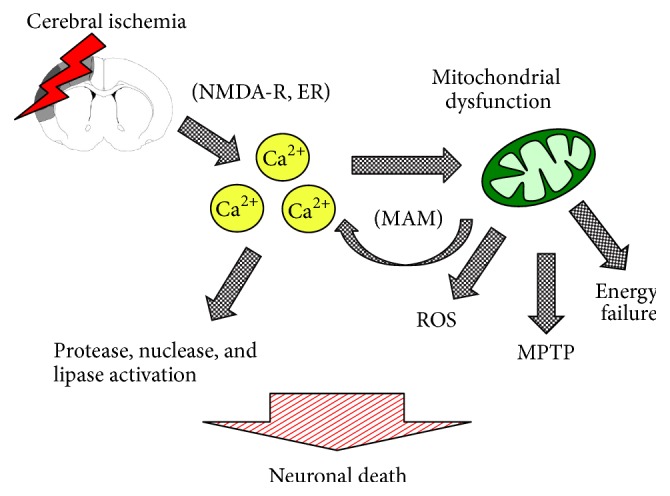
Cerebral ischemia induces mitochondrial dysfunction and neuronal cell death. Ischemia-reperfusion induces elevations in cytosolic Ca^2+^ *via* glutamate binding extrasynaptic NMDA receptors (NMDA-R) and/or mitochondrial-associated membrane (MAM) mediated release from the endoplasmic reticulum (ER). As mitochondrial Ca^2+^ buffering capacity is exceeded and mitochondrial dysfunction ensues, mitochondria produce excessive reactive oxygen species (ROS), decrease capacity for ATP production, and activate the mitochondrial permeability transition pore (MPTP), which can trigger cytochrome c mediated apoptosis. Sustained elevations in cytosolic Ca^2+^ can activate proteases, lipases, and nucleases triggering autophagy or necrotic cell death. NMDA: N-methyl-D-aspartate.

Ischemic stroke remains the most common and debilitating source of cerebral ischemia [1]. However, acute cerebral ischemia can occur* via* a number of mechanisms, including hemorrhagic stroke, subdural and epidural hematoma, subarachnoid hemorrhage, traumatic brain injury, cerebral edema, vascular compression from brain masses, cardiac arrest, or any physiologic condition resulting in low cardiac output. Following cerebral ischemia, astrocytes perform multiple functions beneficial for neuronal survival. One common pathway for neuronal cell death following cerebral ischemia is the accumulation of extrasynaptic glutamate, which triggers mitochondrial dysfunction characterized by imbalances in intracellular Ca^2+^ handling and excessive production of oxidants, eventually leading to neuronal cell death (Figure 1). Astrocytes have been shown to protect neurons from glutamate excitotoxicity during pathophysiologic stresses such as stroke [24], traumatic brain injury [25], and spinal cord injury [26]. However, the astrocyte response to oxidative stress also induces morphologic and phenotypic changes that can paradoxically exacerbate injury, a process termed reactive gliosis or astrogliosis [24, 27]. Reactive astrocytes are identified by increases in cytoplasmic mass and branching processes and increased production of cytoplasmic filaments, most notably glial fibrillary acidic protein. Reactive astrogliosis and subsequent development of an astrocytic scar surrounding the area of injury are essential for isolating the injury site and protecting neurons against harmful substances released from the infarct core. However, this process can also contribute to limiting neuronal regeneration by inhibiting axonal sprouting* via* secretion of chondroitin sulfate proteoglycans [28, 29].

The astrocytic syncytium may also influence neuronal survival by coordinating the spatial delivery of metabolic fuels, thereby maintaining both mitochondrial and cellular integrity. Gap junctions are permeable to both glucose and lactate [30], regulate the development of postinjury edema [31], and have the potential to facilitate delivery of substrates to metabolically active neurons in local areas of decreased perfusion. However, the role of astrocytic gap junctions in stroke remains controversial [32]. For example, astrocytic gap junctions remaining open following ischemia [33] can allow proapoptotic factors and other molecules to spread through the syncytium, expanding the size of the infarct [34]. Moreover, persistently open gap junctions can allow Ca^2+^ waves to propagate throughout the syncytium and induce remote neuronal cell death [35].

Astrocyte-targeted therapies have been shown to protect against neurotoxicity in animal models of neurodegeneration. For example, in astrocytes with an amyotrophic lateral sclerosis- (ALS-) linked mutation in mitochondrial superoxide dismutase (SOD1^G93A^) that disrupts mitochondrial function and results in motor neuron cell death, pretreatment of astrocytes with mitochondrial-targeted antioxidants (ubiquinone and carboxy-proxyl nitroxide coupled to triphenylphosphonium) or with dichloroacetate (DCA, an activator of the pyruvate dehydrogenase complex that improves oxidative phosphorylation coupling) mitigated neuronal cell death in cocultures [36, 37]. Studies specifically targeting astrocytes for improving outcome following cerebral ischemia are limited but have shown promise in rodent models. Augmenting astrocyte extrasynaptic glutamate sequestration by increasing the activity of astrocytic glutamate transporter GLT-1 has been effective at decreasing glutamate excitotoxicity, thereby indirectly maintaining mitochondrial function [38, 39]. However, targeting of the astrocyte response to oxidative stress has also been effective: overexpression of superoxide dismutase 2 (SOD2) in astrocytes reduced evidence of oxidative stress in the hippocampus from transient global ischemia [40] and was also accompanied by preservation of GLT-1. Additionally, increasing astrocytic pyruvate preserved mitochondrial function and improved neuronal survival* via* a glutathione-dependent mechanism [41]. Utilizing direct mitochondrial-targeted approaches in astrocytes may serve to outweigh the negative consequences of reactive gliosis, a necessary astrocyte response for minimizing the degree of injury from cerebral ischemia [42].

### 2.2. Microglia

Microglia constitute 10–15% of all cells in the brain and play an important role in neuronal migration, axonal growth, and synaptic remodeling and in response to ischemic injury (for reviews, see [4, 5, 43]). Microglia share a common myeloid lineage with monocytes and macrophages and similarly act as the primary form of tissue immune defense. The primary functions of microglia are (1) pathogen recognition; (2) phagocytosis of damaged cells, inactive synapses, debris, and infectious agents; and (3) regulation of T-cell responses and induction of inflammation. Under normal physiological conditions, microglia exist in a “resting” state, although they remain highly dynamic with continuous extension and retraction of processes that survey the local microenvironment. Cerebral ischemia induces microglial activation [43], characterized by a change from a ramified to amoeboid shape, loss of branching processes, and production of lysosomes and phagosomes. Mitochondrial function (and dysfunction) appears to play a direct role in microglial activation [44]. Similar to astrocytes, activated microglia have been observed to exert both injurious and protective effects subsequent to cerebral ischemia [45]. This ambiguity has been partially resolved by the observation that microglial activation in response to stroke is a polarized process, described as M1 and M2 activation states (for review, see [5]).

Morphologically, microglia of both M1 and M2 activation states become spherical and retract their processes. Differentiation between the two states is therefore based on antigen expression and cytokine secretion patterns. In M1 or “classical” activation, microglia are characterized by upregulation of proinflammatory surface antigens that can be induced by bacterial lipopolysaccharides or the proinflammatory cytokine interferon-*γ*. M1 activation triggers the production of proinflammatory factors such as tumor necrosis factor-*α* (TNF-*α*), interleukin-1*β* (IL-1*β*), nitric oxide, and reactive oxygen species (ROS), which, in excess, can exacerbate brain injury. TNF-*α* is a critical proinflammatory cytokine released from M1 microglial cells following ischemia, which serves as an activator of receptor-mediated proapoptotic pathways within the neuron, and can further stimulate microglia* via* inducible nitric oxide synthase (iNOS) and cyclooxygenase 2 [46].

Activation of the M2 phenotype by IL-4, IL-10, and/or IL-13 induces surface-receptor expression of several distinct antigens, such as arginase, heparin-binding lectin Ym-1, CD206, and CD36. M2 polarization may also result in a greater capacity for phagocytosis [47–49], important for sequestration of cytotoxic material and in activation of the adaptive immune response. Observations suggest that the time course of polarization and the relative abundance of the two phenotypes depend on the severity, location, and duration of ischemia and reperfusion [43]. Although the precise poststroke temporal kinetics of microglial polarization and mechanisms that determine polarization remain to be determined, current evidence suggests that oxidative stress and mitochondrial function play a central regulatory role [50]. However, more recently, the M1 and M2 classification scheme has been brought into question [51], with the suggestion that, with the advent of novel technologies that better define the complexities of the immunological landscape, the present dichotomy will likely be replaced by a spectrum of activation states that will more accurately reflect the microglial response to ischemic injury.

## 3. Mitochondrial Function in Response to Ischemic Injury

### 3.1. Calcium and Oxidative Stress

Therapeutic strategies that optimize mitochondrial homeostasis may offer a unifying approach to simultaneously target multiple deleterious pathways: mitochondria are central regulators of apoptosis, ROS, and intracellular Ca^2+^ handling. Depletion of energy reserves as occurs during ischemia leads to a massive rise in free cytosolic Ca^2+^ (Figure 1), both from the endoplasmic reticulum (ER) and from the extracellular space, which can then be transmitted to the matrix of mitochondria* via* the mitochondrial-associated membrane (MAM) (for review, see [52]). When mitochondrial matrix Ca^2+^ exceeds buffering capacity, mitochondrial function becomes compromised and results in increased generation of free radicals and formation of the mitochondrial permeability transition pore (MPTP, [53]). Activation and opening on the MPTP can cause release of cytochrome c [54, 55] and other proapoptotic factors into the cytoplasm. The combination of Ca^2+^ accumulation in cytosolic and mitochondrial compartments and excessive ROS levels from reperfusion can induce alterations in protein folding homoeostasis, leading to MAM-mediated amplification in cytosolic Ca^2+^, which can then activate protease, nuclease, and lipase pathways, ultimately contributing to necrotic cell death [52, 56]. Oxidative stress mediated by nitric oxide (e.g., peroxynitrate, ONOO^−^) can also induce reactive gliosis and induce neuronal cell death, an effect mitigated by coadministration of nitric acid synthase (NOS) inhibitors or peroxynitrite scavengers [57].

### 3.2. Glia Mitochondrial Function Regulates Neuronal Survival

**Figure 2:**
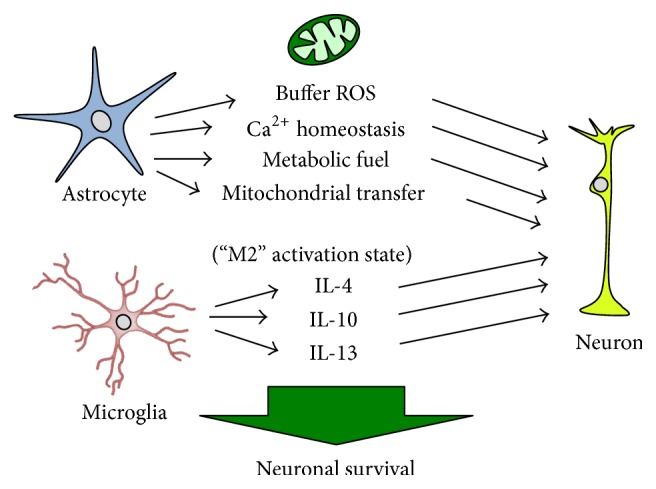
Glia mitochondrial function is essential to neuronal survival following cerebral ischemia. Astrocytes provide protection to neurons by a number of mitochondrial-associated mechanisms, including buffering excessive reactive oxygen species (ROS), maintaining Ca^2+^ homeostasis, and providing metabolic substrate and ATP to neurons. Astrocytes may also regulate neuronal homeostasis and the neuronal bioenergetic response to injury by direct transfer of mitochondria from astrocytes to neurons. Microglial activation polarity determines neuronal fate, with M2 activation state associated with anti-inflammatory cytokine production. Microglial activation is coordinated by microglial and astrocyte mitochondrial function. IL-4: interleukin-4; IL-10: interleukin-10; IL-13: interleukin-13.

Astrocytic mitochondrial function plays several direct and indirect roles in maintaining neuronal survival from ischemic injury (Figure 2). Davis et al. [58] previously observed sequestration of degraded neuronal mitochondria by local astrocytes, suggesting a role for astrocytes in neuronal mitochondrial recycling and biogenesis. More recently, Hayakawa et al. [59] demonstrated that astrocytes are capable of direct transfer of functional mitochondria to neurons and that suppression of this process worsens injury and neurologic outcome from cerebral ischemia. Together, these observations demonstrate a role for mitochondria as a novel medium for neuronal-astrocyte communication and position astrocytes as central to maintenance of neuronal metabolism and bioenergetics in response to cell stress. For example, in addition to coordinating apoptosis, mitochondria are fundamental to maintaining ATP levels* via* oxidative phosphorylation. Neurons, with a relatively higher rate of ATP consumption compared with glia, require a constant source of reducing equivalents to rephosphorylate ATP from ADP and AMP. ATP is required to establish and maintain resting electrochemical gradients and repolarize membranes after depolarization and synaptic transmission and is essential for a host of intracellular signaling and biosynthetic functions. During ischemia, substrate for oxidative phosphorylation (i.e., oxygen and glucose) is reduced, and energy deprivation results in impaired cellular function and eventually cell death (for review, see [60]). Neurons do not normally store glucose as glycogen and must rely on exogenous delivery of substrate [61, 62]. Astrocytes can store glycogen and are therefore critical in maintaining a steady source of metabolic fuel to neurons during ischemic conditions [61, 62]. Lactate generated by astrocytes is transported into neurons* via* the monocarboxylate transporter-2 (MCT-2, [63]), which can serve as a metabolic fuel to maintain basal neuronal activity, particularly when the blood supply of glucose is interrupted [64, 65]. Triggering astrocytic glycolysis is at least in part due to neuronal-astrocyte energy coupling* via* activation of adenosine monophosphate-activated protein kinase (AMPK), an evolutionarily conserved enzyme that functions as an energy sensor by coupling changes in ATP supply to ATP production [66]. Neuronal release of tissue plasminogen activator (tPA), a strong activator of AMPK in astrocytes, induces a switch to glycolysis and subsequent release of lactate, which is then transported into neurons* via* MCT-2 [67]. However, in the nonstressed state, particularly in the poststroke recovery phase when energy requirements are high, a return to oxidative phosphorylation is preferred [60].

Astrocytes are therefore predictably more tolerant to injury than neurons: similar exposure times to oxygen-glucose deprivation (OGD) result in greater injury to primary neuronal cultures* versus* primary astrocyte cultures [68]. Neurons also have limited antioxidant capacity and rely heavily on the antioxidant capacity of astrocyte cytosolic and mitochondrial superoxide dismutase, catalase, glutathione reductase, and glutathione peroxidase to combat oxidative stress [69, 70]. By these and other mechanisms, astrocyte mitochondrial dysfunction can lead to increased neuronal death [70]. For example, disruptions in astrocyte mitochondrial function have been shown to play a direct role in neurotoxicity in animal models of neurodegeneration. Nagai et al. [71] observed that the ALS-linked mutation SOD1^G93A^ was associated with increased neurotoxicity and cell death of motor neurons. Cassina et al. [36] observed that astrocytes with the SOD1^G93A^ mutation demonstrate severe disruptions in oxidative phosphorylation coupling and enhanced mitochondrial superoxide production and recapitulated their neurotoxic effect by pretreatment of astrocytes in neuronal-astrocyte cocultures with mitochondrial inhibitors (rotenone, antimycin A, sodium azide, or fluorocitrate). A further example is that the mitochondrial Ca^2+^ buffering capacity of astrocytes determines astrocyte GLT-1 expression [72], and therefore astrocyte mitochondrial function is intimately tied to the capacity of astrocytes to buffer excessive (extrasynaptic) glutamate and prevent excitotoxicity [73, 74].

In microglia, the role of mitochondrial function in neuronal survival can be considered direct, as neuronal survival is a function of microglial ROS production [75], and indirect,* via* polarization of activation state and subsequent downstream production of cytokines (Figure 2). Cytotoxic M1 activation of microglia is associated with neuronal loss and decline of cognitive and neurobehavioral function [76]. Conversely, M2 activated microglia secrete neurotrophic factors and neuroprotective cytokines. NF-*κ*B, a transcription factor that activates genes regulating cellular survival, growth, differentiation, inflammation, and cell death, plays a central role in regulating the effect of microglia by participating both in protective and in deleterious responses. High concentrations of ROS inactivate NF-*κ*B, inhibiting its binding to DNA, while moderate levels of ROS lead to the sequential phosphorylation, polyubiquitination, and degradation of I*κ*B (inhibitor of *κ*B), allowing activation of NF-*κ*B. Once activated, NF-*κ*B plays a prosurvival role by inhibiting caspase cell death pathways and upregulating transcriptional activation of antiapoptotic proteins and genes involved in decreasing mitochondrial ROS, such as manganese superoxide dismutase [77]. NF-*κ*B also activates antiapoptotic responses regulated by mitochondria, thereby protecting neurons from ischemic brain injury [78]. Therefore, mitochondrial function and microglial activation serve reciprocal roles: targeting cytokines that promote the microglial M2 activation state may result in protecting mitochondrial homeostasis, while direct approaches to augment microglial mitochondrial function may promote M2 activation.

## 4. Hsp70 and Bcl2 in Mitochondrial Homeostasis

Two families of well-known cytoprotective proteins, the Hsp70 family of chaperones and the Bcl2 apoptosis-regulating family, have been shown to be integral to maintaining mitochondrial homeostasis. The Hsp70 family of chaperones is a functionally related group of proteins that assist in the folding or unfolding of proteins, sequestration of denatured proteins, and assembly of protein complexes. Two relevant Hsp70 family members known to regulate glial mitochondrial function are cytosolic Hsp70/Hsp72 and glucose-related binding protein 78 (Grp78)/binding immunoglobulin protein (BiP). The Bcl2 protein family is a central regulator of cell survival by helping to maintain mitochondrial membrane integrity and function and coordinating apoptotic signaling [79, 80].

### 4.1. Bcl2

The Bcl2 family is known to play an important role in the evolution of injury following cerebral ischemia [81]. Overexpressing prosurvival Bcl2 family members protects against cerebral ischemia* in vivo* [82, 83] and* in vitro* [84]. Cytosolic Bcl2 was shown to contribute to MAM formation by localizing to both the ER and the mitochondrial outer membranes [85] and to coordinate ER and mitochondrial Ca^2+^ homeostasis following cerebral ischemia [8, 86]. In addition to regulating induction of apoptosis by controlling mitochondrial outer membrane permeabilization, Bcl2 protein family members have been shown to regulate calcium handling [87] and modulate intercompartmental cross talk between mitochondria and the endoplasmic reticulum [88, 89].

### 4.2. Hsp70/Hsp72

Hsp72, a cytosolic member of the Hsp70 family, participates in protein import and sorting at MAM [90] and regulates downstream Bcl2 expression [91]. Hsp72 is induced in response to a variety of stresses, particularly oxidative stress, and is protective from both necrotic and apoptotic cell death [92, 93].

Hsp72 has been shown to preserve mitochondrial function, reduce oxidative stress, regulate inflammation, and protect from cerebral ischemia [40, 92, 94]. Overexpression protected hippocampus neurons from global cerebral ischemia, mediated in part by increased Bcl2 expression [91]. Overexpression of Hsp72 has been shown to protect both animal and cell models of cerebral ischemia [92]. Astrocyte-specific overexpression of Hsp72 was shown to preserve GLT-1 activity and reduce oxidative stress in the hippocampus following transient global cerebral ischemia [40]. Specifically increasing Hsp72 level in astrocytes demonstrated a reduction in oxidative stress and reduced neuronal vulnerability to global cerebral ischemia [40], as well as improving long-term recovery following focal cerebral ischemia [95]. Evidence suggests that Hsp72 also modulates inflammation from cerebral ischemia [96]. Regulation of NF-*κ*B by Hsp72 can occur* via* inhibition of I*κ*B phosphorylation by IKK and NF-*κ*B dissociation [97] and* via* binding IKK to impair NF-*κ*B signaling [98]. Increased levels of Hsp72 have been shown to decrease the negative effects of NF-*κ*B activation in astrocytes* via* reductions in iNOS expression [98]. Activation of NF-*κ*B was inhibited significantly in Hsp72-overexpressing microglia and transgenic mice [97].

### 4.3. Grp78

Grp78, a regulator of the ER unfolded protein response [52, 99], is largely localized to the endoplasmic reticulum [100] but has been shown to translocate to mitochondria [101], suggesting a role in MAM-dependent Ca^2+^ transport between ER and mitochondria. Studies utilizing a green fluorescent/Grp78 fusion protein reported targeting to mitochondria within a short period of ischemia-like stress [86]. Overexpression of Grp78 in BV2 mouse microglial cell lines [102] and astrocytes protected against ischemic injury and preserved respiratory activity and mitochondrial membrane potential after ischemic stress [103]. Pharmacological induction of Grp78 reduced neuronal loss in both forebrain and focal cerebral ischemia [104, 105]. Grp78 appears to play an important role in ischemia associated with oxidative stress: increased levels of Grp78 were observed in astrocytes and microglia associated with overproduction of ROS [106, 107].

In addition to effects on oxidative stress and mitochondrial function, Grp78 also mediates the inflammatory response. Grp78 was shown to stimulate the production of the anti-inflammatory cytokines IL-4 and IL-10 through specific T lymphocytes [108, 109]. Interestingly, IL-10 deprivation in mice brains also induced Grp78 expression [110], suggesting a negative-feedback mechanism. Conversely, lower levels of Grp78 inhibited upregulation of IL-6 under glucose-deprived conditions [111]. Though the precise regulatory role of Grp78 in the inflammatory cascade remains to be elucidated, the critical role of astrocytes and microglia in the neuroinflammatory response to cerebral ischemia suggests that pharmacologic manipulation of Hsp72 family members in glia may be a powerful therapeutic approach.

## 5. MicroRNAs Regulate the Mitochondrial Response to Ischemia

Studies investigating the role of miRs in cerebral ischemia have largely focused on changes in miR expression patterns with ischemia [112]. To define the role of a given miR of interest, predicted molecular targets that have complementarity to the binding sequence of the miR are identified using a bioinformatic approach. Efficacy of translational inhibition can be tested when the 3′UTR of the putative target mRNA is placed downstream of a luciferase reporter construct. Using this approach, two brain-enriched miRs, miR-181a and miR-29a, were identified as important mediators in the evolution of injury and in determining outcome following stroke (see [113]). A recent microarray analysis of miR expression in the four principal cell types of the CNS (neurons, astrocytes, oligodendrocytes, and microglia) delineated a preferential cellular expression pattern of individual miRs [114]. Notably, miR-181a and miR-29a were more highly expressed in astrocytes, corroborating previous observations [115, 116].

### 5.1. miR-181

The brain-enriched miR-181 family contains four highly conserved members, miR-181a, miR-181b, miR-181c, and miR-181d, which play a role in mitochondrial function, redox state, and inflammatory pathways [7]. Bioinformatics and dual luciferase assays were used to identify and validate miR-181a as a regulator of several Hsp70 [117, 118] and Bcl2 family members [118]. Increased miR-181a exacerbated injury both* in vitro* and* in vivo*, while reduced levels were associated with reduced injury and increased Grp78 protein levels [118]. Overexpression of miR-181a in astrocytes enhanced disruption of mitochondrial membrane potential and increased ROS formation and cell death from glucose deprivation [118]. Intracerebroventricular infusion and intravenous injection of 181a antagomir, a chemically modified 181a inhibitor optimized for* in vivo* use, were used to treat mice after ischemic injury. miR-181a antagomir was effective at abolishing endogenous expression of miR-181a and showed substantial neuroprotective effects against ischemic neuronal damage and neurological impairment in mice. This protective effect, including recovery of motor function and coordination, persisted over 28 days [119], concordant with decreased expression of Bcl2 and X-linked inhibitor of apoptosis protein (XIAP). Interestingly in primary neurons miR-181a failed to significantly alter levels of Bcl2 and did not improve survival after ischemia-like injury [120]. The difference in effects of miR-181a suppression between different brain cell types may be the result of differences in baseline levels of expression or changes in expression in response to ischemia.

Another member of this family, miR-181c, was identified as directly targeting tumor necrosis factor-alpha (TNF-*α*) following ischemia, thereby regulating microglial activation and microglial-mediated neuronal injury [121]. Ectopic expression of miR-181c was also shown to suppress expression of iNOS, leading to decreased production of NO following OGD [121]. Additionally, the microglial activator Toll-like receptor 4 (TLR4) was shown to be a target of miR-181c in microglial cells. miR-181c inhibited NF-*κ*B activation induced by OGD and the downstream production of proinflammatory mediators by suppressing TLR4 expression [122].

### 5.2. miR-29

The miR-29 family, composed of miR-29a, miR-29b, and miR-29c, is distributed across the central nervous system and enriched in astrocytes [123, 124]. All members have been shown to regulate various facets of inflammation [125]. Inhibition of miR-29b significantly reduced expression of activated microglial proinflammatory mediators such as TNF-*α*, IL-1b, IL-6, and monocyte chemoattractant protein-1 [126]. Recently, miR-29b has been recognized as a survival factor in neuronal cells by silencing the proapoptotic BH3-only family [127]. Interestingly, Shi et al. reported that increasing miR-29b had the effect of* promoting* neuronal cell death in focal ischemia by inhibiting Bcl2l2 (protein BCL-w), an antiapoptotic member of the Bcl2 protein family [128]. Whether the same effect occurs in astrocytes or microglia has not been investigated; however, computational algorithms (i.e., TargetScan5.1, http://www.targetscan.org/) predict that miR-29b targets several members of the Bcl2 family known to be both protective and harmful. The multiple functions of miR-29b are therefore likely due to which Bcl2 family member exerts the more dominant effect in a given cell type and in response to a given injury paradigm.

Ischemic stroke induced by middle-cerebral artery occlusion (MCAO) in mice caused loss of miR-29b and higher 12-lipoxygenase activity in infarcted tissue [129]; delivery of miR-29b mimic markedly attenuated the infarct size. Ouyang et al. observed that miR-29a expression significantly increased in the more ischemia-resistant hippocampal subregion dentate gyrus but decreased in the more ischemia-sensitive cornu ammonis 1 subregion following transient global ischemia [115]. In the setting of* in vitro* ischemia, Ouyang et al. observed that miR-29a mimic protected and miR-29a inhibitor aggravated astrocyte injury and mitochondrial function by targeting the Bcl2 family member p53 upregulated modulator of apoptosis (PUMA, [115]). PUMA binds and antagonizes all known antiapoptotic Bcl2 family members and activates two key multidomain proapoptotic Bcl2 family proteins, BAX and BAK, leading to mitochondrial dysfunction and caspase activation [130]. Most recently [131], we observed in astrocytes cultured from CA1 and DG hippocampal subregions that CA1 astrocytes exhibited more cell death and a greater decrease in miR-29a subsequent to glucose deprivation injury. We identified the mitochondrial voltage-dependent cation channel 1 (VDAC1) as a target of miR-29a in these astrocytes. Located in the outer mitochondrial membrane, VDACs mediate intercompartmental transport of anions, cations, and ATP between the cytosol and mitochondria (for review, see [132]). VDAC1 is the most abundantly expressed and is thought to regulate mitochondrial function and cell survival in response to injury [133]. We observed that increasing miR-29a in CA1 astrocytes decreased VDAC1 and improved cell survival, while knockdown of VDAC1 improved survival. Moreover, the protective effect of miR-29a was eliminated by inhibition of miR-29a/VDAC1 binding* via* cotreatment with a target-site blocker. Together, these findings suggest that the selective vulnerability of the CA1 to injury may be due in part to a limited miR-29a response in CA1 astrocytes, thereby allowing a greater increase in VDAC1-mediated cellular dysfunction in CA1 astrocytes.

## 6. Conclusions and Future Directions

As targets for stroke therapy, miR-based strategies provide the advantage of rapid onset of action, a critical element in developing effective clinical treatments for stroke, and endogenous miR expression levels can be pharmacologically manipulated. A successful phase 2 trial of the first miR-targeted drug, a locked nucleic acid targeting miR-122 to treat hepatitis C, has recently been completed [134], demonstrating that rapid translation of miR-based therapies from bench to clinic may be possible once candidate targets are identified.

**Figure 3:**
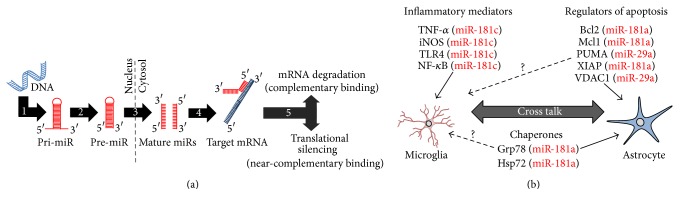
MicroRNAs (miRs) regulate mitochondrial function in glia. (a) miR biogenesis begins in the nucleus with genomic transcription of pri-miR (1). Drosha-mediated cleavage results in pre-miR (2), which is then exported to the cytosol by Exportin-5 and processed to the final mature miR forms by Dicer (3). In the cytosol, either the leading or the reverse complementary mature miR strand is then free to interact with the 3′ untranslated region of target messenger RNAs (mRNAs, (4)). miR/mRNA complexes are then targeted by the RNA-induced silencing complex (5) for either mRNA degradation or translational silencing, depending on the degree of miR/mRNA binding complementarity. (b) miR-mediated control of microglial mitochondrial function and activation state occurs secondary to miR targeting of cytokines and inflammatory mediators. miRs directly affect mitochondrial function in astrocytes by targeting Bcl2 family members and chaperones. Whether the same miR targets are relevant in microglia has not yet been determined (dashed arrows), yet astrocyte/microglial cross talk suggests at least an indirect role. Bcl2: B-cell lymphoma 2; DNA: deoxyribonucleic acid; Grp78: glucose-related protein 78; Hsp75: heat shock protein 75; iNOS: inducible nitric oxide synthase; Mcl1: myeloid cell leukemia 1; NF-*κ*B: nuclear factor kappa B; PUMA: p53 upregulated modulator of apoptosis; TLR4: Toll-like receptor 4; TNF-*α*: tumor necrosis factor-alpha; VDAC1: voltage-dependent anion channel 1; XIAP: X-linked inhibitor of apoptosis protein.

Astrocytes and microglia play critical roles in neuronal survival following stroke and are ideal cellular targets for novel therapeutic approaches. miRs target proteins directly involved in maintaining astrocyte mitochondrial homeostasis in response to stress and neuroinflammatory mediators that regulate microglial activation with downstream effects on mitochondrial function (Figure 3). Recent findings [58, 59] demonstrating a novel role for mitochondria as a potential transduction pathway for neuronal-astrocyte cross talk and the emerging relevance of astrocytes as regulators of the neuronal bioenergetic response to cell stress [59] suggest that future studies investigating the role of miRs in these processes may provide a novel angle to overcome prior translational hurdles in the search for new clinical therapies for cerebral ischemia. Moreover, microglial activation can depend on astrocytic release of ATP in response to local injury, suggesting that astrocyte mitochondrial function plays a direct role in microglial activation state [24]. Therefore, miR-based therapeutic interventions targeting mechanisms that mediate cross talk between astrocytes and microglia may provide an alternative approach to simultaneously coordinate both glial mitochondrial function and microglial activation polarization.

The short (only 5–7-nucleotide-long) sequence in the mature miR determines binding specificity to target mRNAs; as a consequence, a single miR can bind multiple mRNAs, and a single mRNA can be bound by multiple miRs, creating a new and complex layer of posttranscriptional control. Identifying glial-enriched miRs that target multiple mitochondria-regulating genes and gene families may produce a substantial effect* versus* single gene silencing techniques, for example, with small interfering RNA or short hairpin RNA. However, the combinatorial effect of the short binding sequence can also offset any protective effects if the same miR simultaneously binds gene family members with opposite functions, as has been demonstrated with miR-29 and Bcl2 family members. Therefore, it remains critical to identify glial mitochondrial targets, test and verify the predicted miR/mRNA interaction, and investigate the effect of varying miR levels both* in vitro* and* in vivo* on mitochondrial function and injury in response to cerebral ischemia.
